# Full-length human cytomegalovirus terminase pUL89 adopts a two-domain structure specific for DNA packaging

**DOI:** 10.1371/journal.ppat.1008175

**Published:** 2019-12-06

**Authors:** Janine Theiß, Min Woo Sung, Andreas Holzenburg, Elke Bogner

**Affiliations:** 1 Institute of Virology, Charité - Universitätsmedizin Berlin, Berlin, Germany; 2 Department of Biochemistry and Molecular Biology, Oregon Health and Science University, Portland, Oregon, United States of America; 3 Department of Molecular Science, School of Medicine, The University of Texas Rio Grande Valley, Brownsville-Edinburg-Harlingen, Texas, United States of America; State University of New York Upstate Medical University, UNITED STATES

## Abstract

A key step in replication of human cytomegalovirus (HCMV) in the host cell is the generation and packaging of unit-length genomes into preformed capsids. The enzymes involved in this process are the terminases. The HCMV terminase complex consists of two terminase subunits, the ATPase pUL56 and the nuclease pUL89. A potential third component pUL51 has been proposed. Even though the terminase subunit pUL89 has been shown to be essential for DNA packaging and interaction with pUL56, it is not known how pUL89 mechanistically achieves sequence-specific DNA binding and nicking. To identify essential domains and invariant amino acids vis-a-vis nuclease activity and DNA binding, alanine substitutions of predicted motifs were analyzed. The analyses indicated that aspartate 463 is an invariant amino acid for the nuclease activity, while argine 544 is an invariant aa for DNA binding. Structural analysis of recombinant protein using electron microscopy in conjunction with single particle analysis revealed a curvilinear monomer with two distinct domains connected by a thinner hinge-like region that agrees well with the predicted structure. These results allow us to model how the terminase subunit pUL89’s structure may mediate its function.

## Author summary

HCMV is a member of the herpesvirus family and represents a major human pathogen causing severe disease in newborns and immunocompromised patients for which the development of new non-nucleosidic antiviral agents are highly important. This manuscript focuses on DNA packaging, which is a target for development of new antivirals. The terminase subunit pUL89 is involved in this process. The paper presents the identification of DNA binding and nuclease motifs with invariant amino acids and highlights its first 3-D surface structure at approx. 3 nm resolution. At this resolution, the calculated 3-D surface structure matches well with the predicted structure. In conjunction with earlier studies it was possible to define structure-function relationships for the HCMV terminase subunit pUL89.

## Introduction

The human cytomegalovirus (HCMV) genome consists of a double stranded linear DNA that immediately circularizes after nuclear import [1, 2]. The HCMV genome is approximately 235 kb in size, containing repetitive and unique components. Viral replication of this covalently closed circular DNA leads to the formation of concatemers, which must be resolved into unit-length genomes during packaging [3, 4]. The viral DNA is translocated into the capsid through the portal vertex. A group of specific enzymes called terminases, first described for double-stranded DNA bacteriophages [5], mediates cleavage and by binding to the portal during packaging of viral DNA. Terminases represent multifunctional heterooligomers in which one subunit provides ATP for translocation into preformed capsids and cleaves concatemers into unit-length genomes while the other is required for sequence specific DNA binding [5–8]. Caudovirales and Herpesviridae share a common ancestry that is reflected in similar capsid structures and terminases. Similarities in sequence specific cleavage are also found in Herpesviridae and cos-containing dsDNA bacteriophages (e.g. λ) [9].

In HCMV, the terminase consists of the subunit pUL89, the subunit pUL56 and a hypothetic third subunit, pUL51 [10–14]. The terminase subunits are essential for virus replication and highly conserved throughout the herpesvirus family [15, 16].

The DNA packaging process requires site-specific binding and cleavage at pac motifs located within the *a* sequence of the terminal repeats and in opposite orientation between the L- and S-segment [17, 18]. The terminase subunit pUL56 (i) mediates specific binding to packaging elements on the concatemers, (ii) transduces energy for translocation of the DNA to the procapsids and (iii) associates itself with the capsid to enable the entry of the DNA [19–21]. The terminase subunit pUL89 is thought to be required for cleavage of concatemers into unit-length genomes in the cleavage/packaging process [22]. The HSV-1 terminase subunit pUL15 has similar functions and is essential for viral genome packaging [6]. Whether this cleavage is mediated by site-specific duplex nicking or not is unknown, but it is a prerequisite for packaging.

In the last decade, terminases of bacteriophages and herpesviruses have been intensively studied. Although numerous reports have yielded insights into the function of the terminases, the structural requirements are not completely understood. Analyses of the crystal structures of the phage T4 large terminase subunit and of phage SPP1 G2P nuclease domain demonstrated that the structure of the DNA binding surface during translocation is not conserved [23, 24]. The structure of the nuclease domains of HCMV pUL89 [25] and recently the homolog of HSV-1 pUL15 [26] were solved by crystallography. Both structures showed a core fold resembling that of RNAse H [26]. Structural studies of the HCMV terminase have been hampered by the lack of sufficient quantities of the protein as the preparations suffered from aggregation and minimal yields. The latter is likely due to its toxicity to the heterologous host (baculovirus). To this end, single particle analysis of negatively stained specimens presented itself as one of the few feasible ways forward.

Here we report low resolution 3-D structure of full-length HCMV pUL89 arranged as a two-domain monomer incorporating sites involved in DNA binding and nuclease activity. By alanine scanning mutagenesis of purified pUL89 a specific nuclease motif as well as a motif crucial for DNA binding were identified. A first model of structure-function relationship for pUL89 is hereby presented.

## Results

### In silico analysis of the complete pUL89 structure

**Fig 1:**
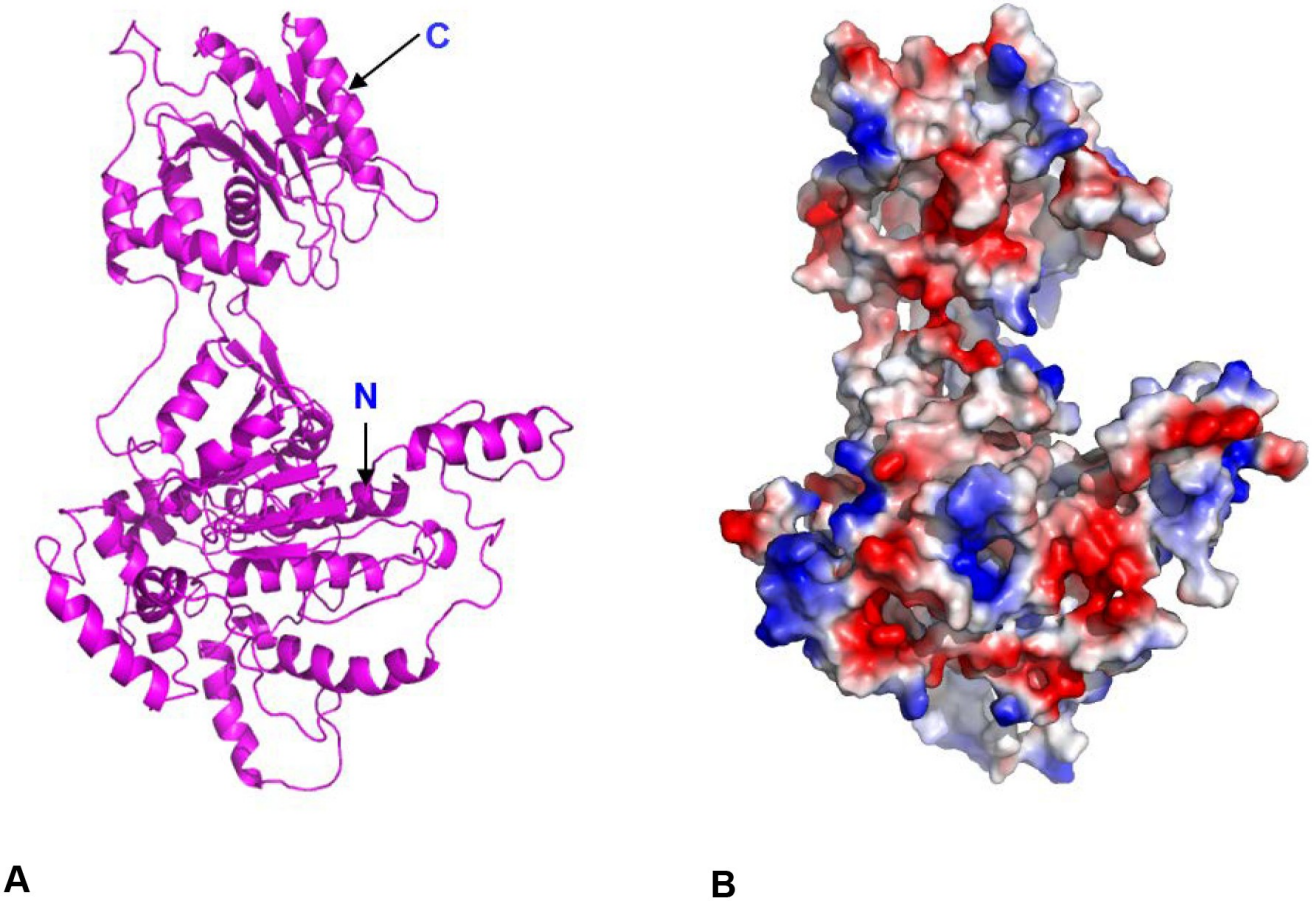
Structural prediction of full-length pUL89. (A) The ribbon representation of full-length pUL89 model. (B) Surface charge representation of full-length pUL89 is shown. Region of positive, negative and neutral electrostatic potential are indicated in blue, red and white, respectively. Arrows indicate the N and C-terminus.

In order to gain further structural insights into putative regions of the HCMV terminase subunit pUL89, *in silico* analysis of the structure was performed using Phyre2 in intensive mode [27]. The analysis took more than 100 structures of homologous proteins (S1 Fig) into account and led to a model of N-Terminal and C-Terminal halves separated by a flexible structure. The crystal structure of the homologous proteins from bacteriophage T4 gp17 (PDB: 3EZK_B), deep-sea thermophilic phage D6E large terminase (PDB: 5OE9_C) and bacteriophage Sf6 gp2 (PDB: 4IDH_A) showed a similar organisation. The best alignment with the maximum confidence was obtained with T4 gp17, D6E TerL and for the last 279 aa with nuclease domain of pUL89 (PDB: 3N4P) (S2 Fig). All these predictions suggested that pUL89 is the TerL of HCMV. A scheme of the predicted structure of pUL89 is shown in Fig 1.

Protein substitutions in the following analyses were chosen on the basis of electrostatic potential of the surface of pUL89 in order to identify motifs for nuclease activity and DNA binding (Fig 1B). In addition, sequence alignment of five homologous proteins using the program Clustal Omega demonstrated that most of the amino acids of the putative motifs are highly conserved (S3 Fig).

### Purification of full-length pUL89

**Fig 2:**
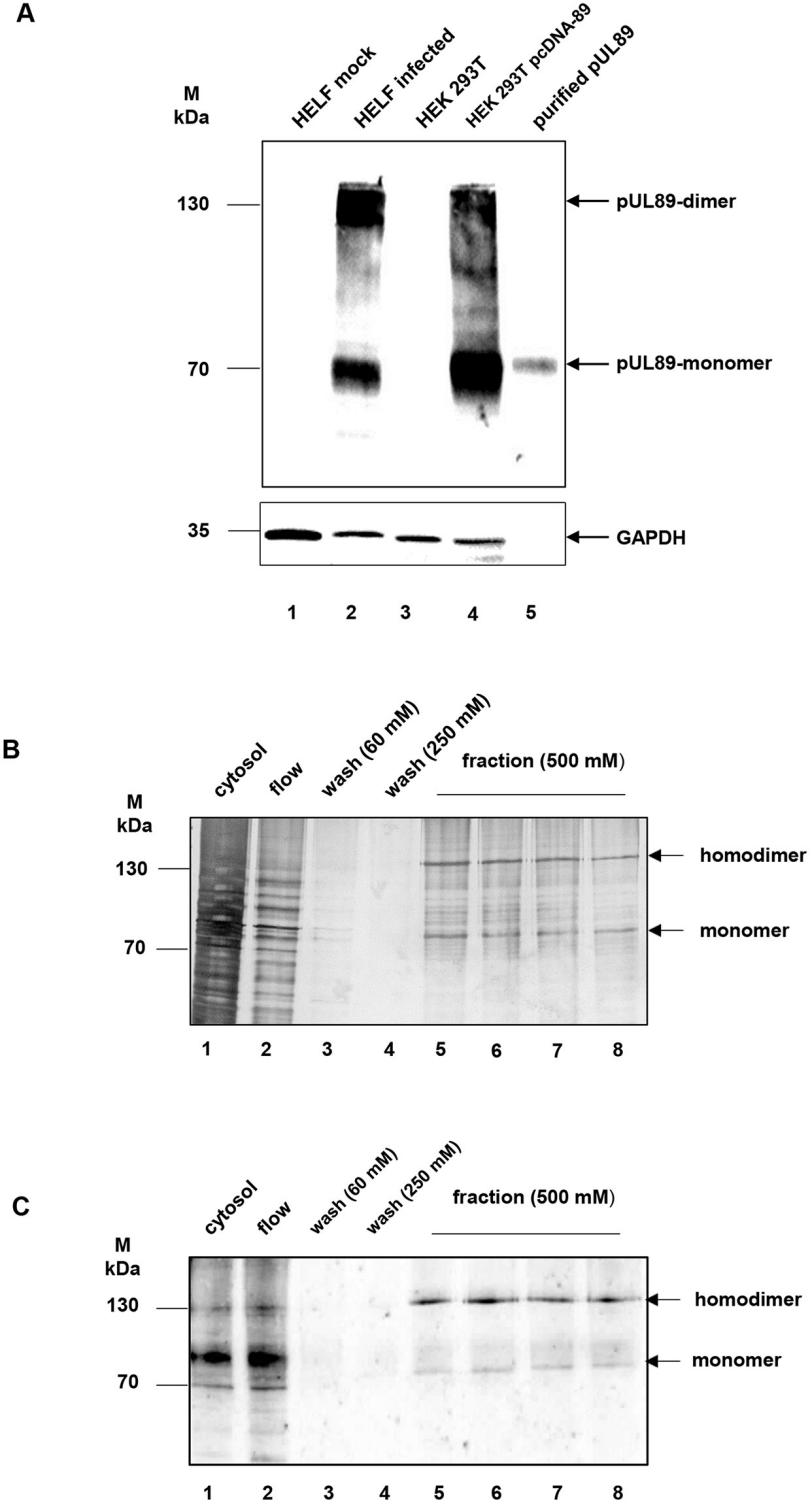
Purification of pUL89 from transfected HEK 293T cells. (A) Characterization of the monoclonal antibody against pUL89 (mAbUL89). Mock-infected (lane 1) and infected HELF (lane 2), HEK 293T (lane 3), HEK 293T transfected with pcDNA-89 (lane 4) and purified pUL89 (lane 5) were separated by 10% SDS–PAGE and transferred to nitrocellulose. The immunoblot was reacted with mAbUL89. GAPDH served as a loading control. (B) Silver staining of purification of pUL89. Lane 1, cytosol; lane 2, flow; lane 3–4, wash fractions (60 mM and 250 mM imidazole), lane 5–8 fraction with eluted pUL89 (500 mM imidazole). (C) Immunoblot of purification of pUL89. Lane 1, cytosol; lane 2, flow; lane 3–4, wash fractions (60 mM and 250 mM imidazole), lane 5–8 fraction with eluted pUL89 (500 mM imidazole). Probes were subjected to SDS-Page followed by silver staining or immunoblot analysis with anti-pUL89 (mAbUL89). Markers (kDa) are indicated on the left, the position of pUL89 on the right.

To determine the functional motifs of pUL89 it was important to use purified protein mutants in the cleavage and binding assay. For this analysis, a eukaryotic expression system for protein purification was chosen. Human embryonic kidney cells (HEK-cells) were transiently transfected with the plasmid pcDNA-UL89 or mutant plasmids. The recombinant proteins contain a His_6_-tag that enables protein purification with immobilized metal ion affinity chromatography (IMAC), using His Mag Sepharose Ni (Fig 2B). Silver staining revealed two distinct proteins of approximately 130 and 70 kDa in the eluted fractions (Fig 2B, lane 5–8). Immunoblot analysis using a monospecific antibody against pUL89, mAbUL89 (Fig 2A), confirmed that both proteins represent the homodimer (130 kDa) and the monomer (70 kDa) of pUL89 (Fig 2C, lane 5–8).

### Identification of the putative nuclease domain of full-length HCMV pUL89

In order to identify essential amino acids for the nuclease activity purified pUL89 was analysed in the cleavage assay in conjunction with 1D gel quantification analysis.

To determine the amount of pUL89 that is sufficient for nuclease activity, analysis were performed with circular covalently closed (S4A Fig) and linearized plasmid DNA (S4B Fig). Concentrations from 0.4 μM pUL89 is sufficient to convert the ccc DNA to open circular molecules with retarded migration and to linear molecule (S4A Fig, lane 6). In contrast, for nuclease activity on linearized DNA, target concentrations >1.0 μM were required (S4B Fig, lanes 3–11). In the following analysis 0,5 μM of pUL89 was used.

**Fig 3:**
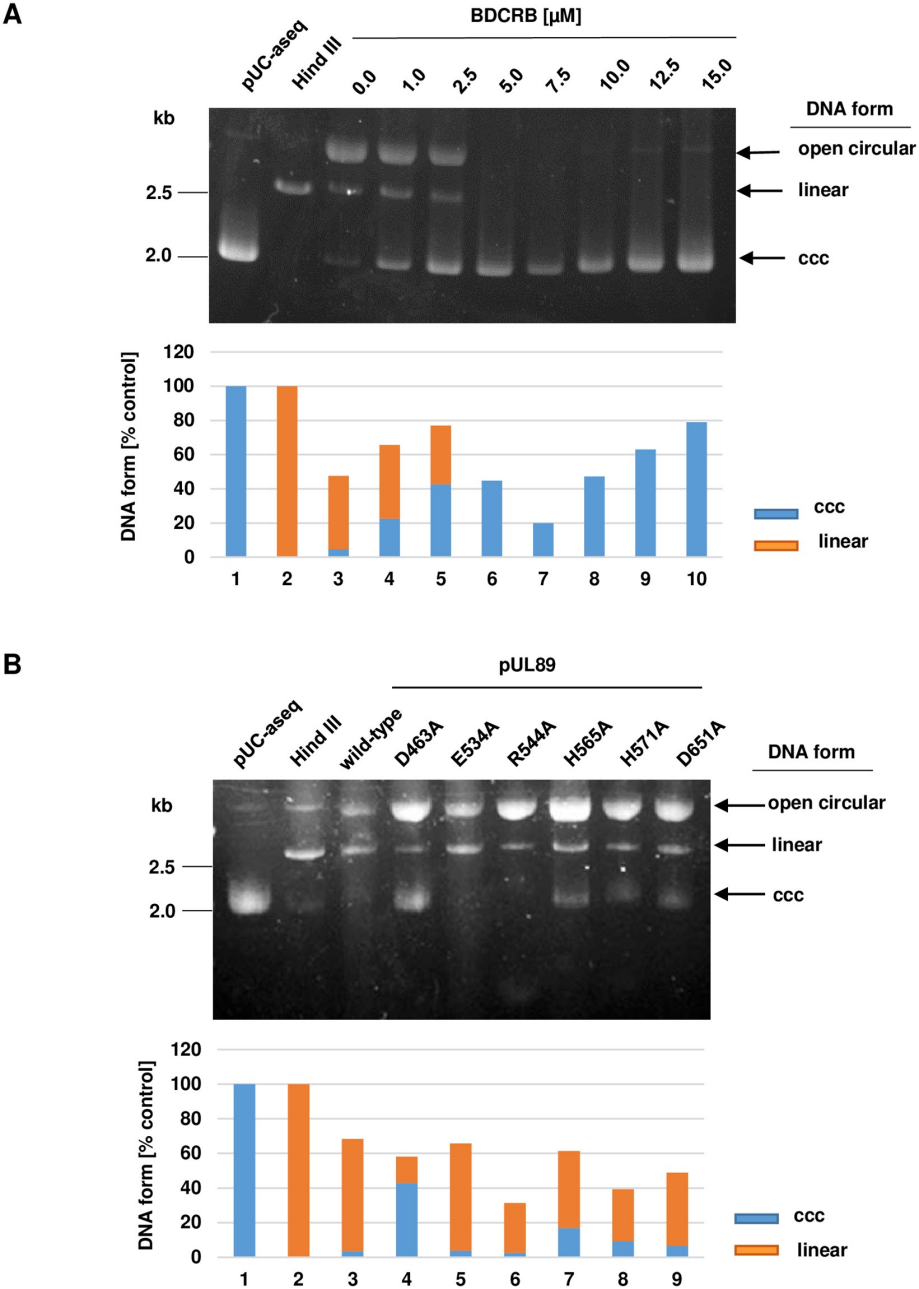
Nuclease activity of wild-type and mutant pUL89 proteins. (A) Purified pUL89 (0.5 μM) together with treatment with BDCRB (0–15μM) and (B) purified wild-type pUL89 (0,5 μM) and mutants of pUL89 (0,5 μM; pUL89-D463A; pUL89-E534A; pUL89-R544A; pUL89-H565A; pUL89-H571A; pUL89-D651A) were analysed by nuclease activity assay. (A) Lane 1, 600 ng pUC-aseq; lane 2, incubation with restriction endonuclease Hind III, lane 3 incubated with pUL89, lane 4, incubated with pUL89 treated with 1.0 μM BDCRB; lane 5, incubated with pUL89 treated with 2.5 μM BDCRB; lane 6, incubated with pUL89 treated with 5.0 μM BDCRB; lane 7, incubated with pUL89 treated with 7.5 μM BDCRB; lane 8, incubated with pUL89 treated with 10 μM BDCRB; lane 9, incubated with pUL89 treated with 12.5 μM BDCRB; lane 10, incubated with 0.5 μM pUL89 treated with 15 μM BDCRB. (B) Lane 1, 600 ng pUC-aseq; lane 2, incubation with restriction endonuclease Hind III, lane 3, incubated with wild-type pUL89, lane 4, incubated with pUL89-D463A; lane 5, incubated with pUL89-E534A; lane 6, incubated with pUL89-R544A; lane 7, incubated with pUL89-H565A; lane 8, incubated with pUL89-H571A; lane 9, incubated with pUL89-D651A. After incubation with plasmid DNA at 37°C, all probes were treated with proteinase K (final concentration 1 μg/μl) at 65°C. The arrows indicated three different plasmid DNA forms: circular covalently closed molecules (ccc), open circular molecules and linear forms. The quantifications were performed with the software Phoretix 1D (BioSytematica) and shown below the image.

First, we tested the impact of the terminase inhibitor BDCRB [22, 28]). Separation by agarose gel electrophoresis showed that pUC-aseq [22] bearing a single *a* sequence incubated in the absence of pUL89 were present as a fast migrating circular covalently closed (ccc) form (Fig 3A, lane 1). After treatment with restriction endonuclease Hind III, which recognizes a single restriction endonuclease site on pUC-aseq, the ccc form was converted to a linear molecule (Fig 3A, lane 2). In the presence of pUL89, plasmid DNA was converted to open circular molecules with retarded migration and then to linear forms (Fig 3A, lane 3). Concentrations of the inhibitor ranging from 1.0 μM (22.6% ccc, 43.0% linear) to 2.5 μM (42.7% ccc, 34.3% linear) showed a significant reduction of the nuclease activity (Fig 3A, lanes 4–5). In the presence of 5.0 μM to 15.0 μM BDCRB complete inhibition of nuclease activity was observed (0% linear forms; Fig 3A, lanes 6–10). Thus the terminase inhibitor BDCRB could block the nuclease activity of purified pUL89.

In the following experiments, all purified pUL89 mutants were incubated with plasmid pUC-aseq. In the presence of purified pUL89 (wild-type), pUL89-E534A and pUL89-R544A DNA was efficiently converted to open circular molecules with retarded migration and to linear forms (3.5%, 3.9% and 2.5% ccc; Fig 3B, lanes 3, 5–6). In the presence of pUL89-H565A, pUL89-H571A and pUL89-D651A, plasmid DNA was converted to open circular molecules and to linear forms (Fig 3B, lanes 7–9). However, the presence of approximately 12% of the ccc molecules indicate that the nuclease activity was slightly reduced. In contrast, pUL89-D463A does have the most significant effect on the nuclease activity (Fig 3B, lane 4). 42.9% of the DNA of this mutant are ccc molecules. These results demonstrated that aspartate 463 is required for the nuclease activity of pUL89.

### Identification of the putative DNA binding domain of full-length pUL89

In order to identify DNA binding motifs, colorimetric DNA binding assays were performed with purified wild-type pUL89 or mutants of pUL89 and biotinylated ds DNA probes. This assay is based on two strong interactions: (i) binding of his-tagged pUL89 to nickel-coated microplates and (ii) binding of biotinylated DNA probe to avidin. The avidin is conjugated to HRP that catalyses a colorimetric reaction. This sensitive detection enables quantification.

**Fig 4:**
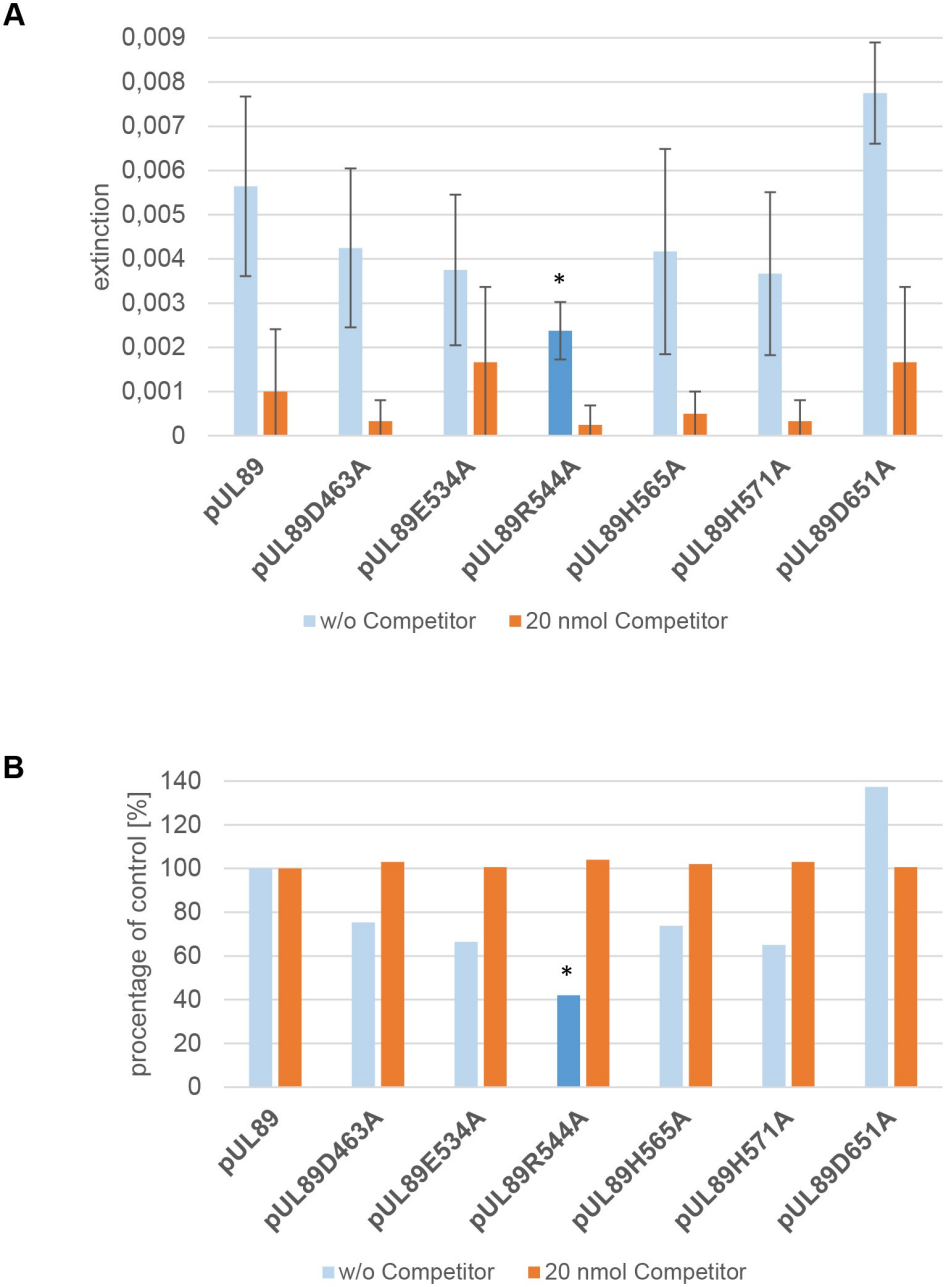
Identification of amino acids required for DNA binding using colorimetric DNA binding assays. The assays were performed with purified pUL89 or indicated purified mutants (pUL89-D463A; pUL89-E534A; pUL89-R544A; pUL89-H565A; pUL89-H571A; pUL89-D651A) and biotinylated DNA probe. (A) Extinction at 620 nm. Lane 1, pUL89 wild-type (control) and values in the presence of 20 nmolar excess of competitor; lane 2, pUL89-D463A, +/- 20 nmolar excess of competitor; lane 3, pUL89-E534A, +/-20 nmolar excess of competitor; lane 4, pUL89-R544A, +/- 20 nmolar excess of competitor; lane 5, pUL89-H565A,+/-20 nmolar excess of competitor; lane 6, pUL89-H571A +/-20 nmolar excess of competitor; lane 7, pUL89-D651A, +/- 20 nmolar excess of competitor. (B) Percentage of reduction of DNA binding in comparison to the control. Lane 1, pUL89 wild-type (control), +/- competitor; lane 2, pUL89-D463A, +/- competitor; lane 3, pUL89-E534A, +/- competitor; lane 4, pUL89-R544A, +/- competitor; lane 5, pUL89-H565A, +/- competitor; lane 6, pUL89-H571, +/- competitor; lane 7, pUL89-D651A, +/- competitor. Values represent mean ± SD from three independent experiments. * *p*-value ≤ 0.05.

The results show that DNA binding of mutant pUL89-D651A was not affected, while the mutants pUL89-D463A, pUL89-H656A, pUL89-E534A and pUL89-H571A showed a DNA binding activity of approximately 70% (Fig 4). In contrast, only the mutant pUL89-R544A, show a significant reduction of 57.91% of DNA binding (Fig 4). In conclusion, these results demonstrated that the arginine 544 of pUL89 plays an important role in DNA binding.

**Fig 5:**
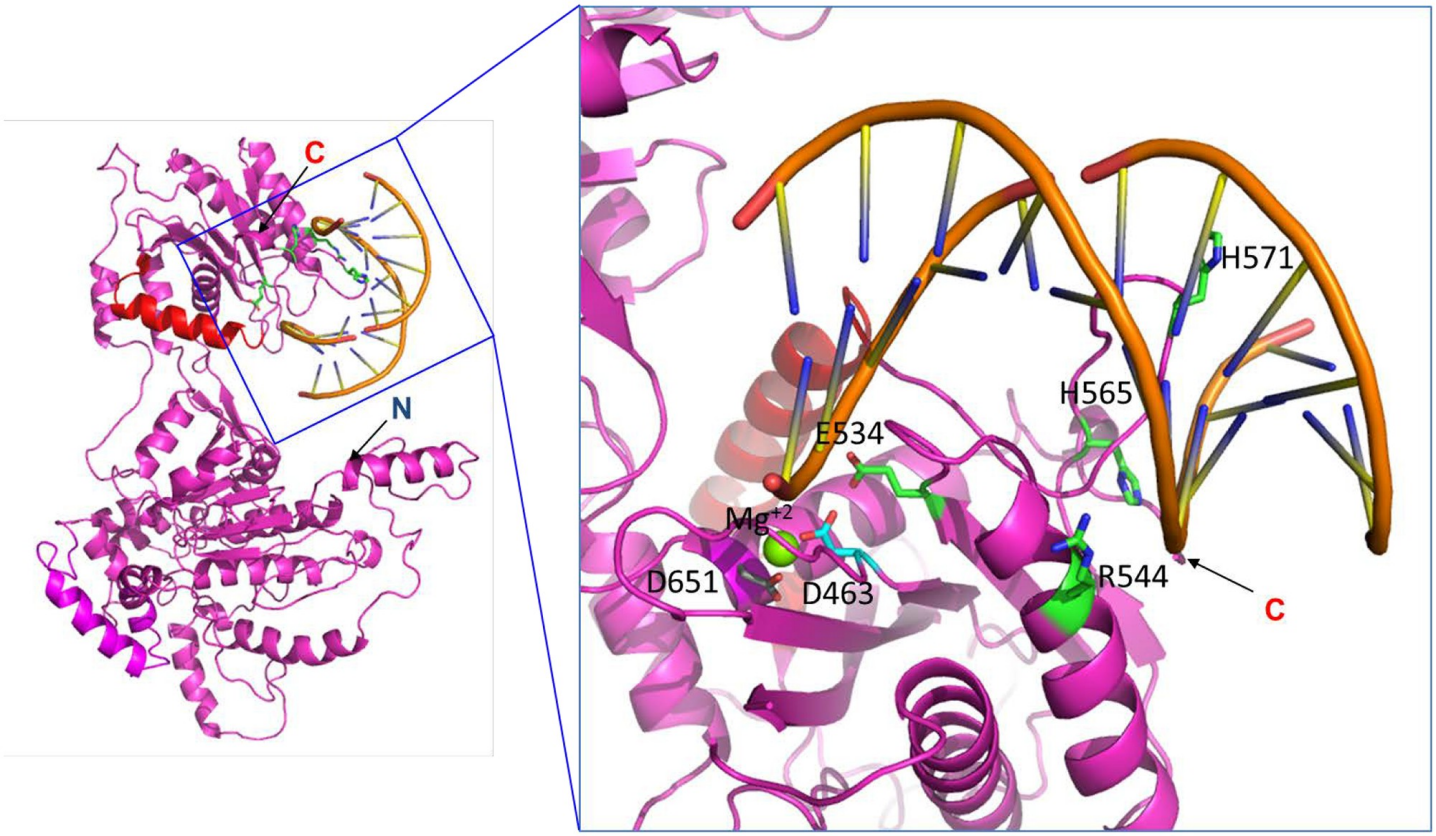
Predicted structure of the DNA binding motif of full-length wild-type pUL89. The left figure shows a ribbon representation of full-length pUL89 and double stranded DNA. Residues 580–600, expected to be responsible for interaction with pUL56, are colored in red. The figure on the right represents a magnified view of the local structure from the boxed region highlighting the predicted functional motifs and including high-resolution data for the C-terminal part of pUL89 (Nadal et al.; 25). Nadal et al. (2010) identified the nuclease domain comprised of D463, E534 and D651 and alluded to a Mg2+ dependent mechanism (Mg2+ is shown in green). In contrast to this, by using the full-length pUL89, this report confirms D463 (cyan) as an amino acid required for nuclease activity and argine 544 as critical for binding double stranded DNA (shown in green).

The results of the nuclease activity as well as DNA binding analysis are illustrated concerning structure-function relationships (Fig 5). The boxed region shows the predicted functional motifs in the presence of DNA. Argine 544 (green) is in close proximity to the DNA while aspartate 463 (cyan), required for nuclease activity, is in close proximity of Mg^2+^ (green).

### Single particle analysis of baculovirus expressed protein

**Fig 6:**
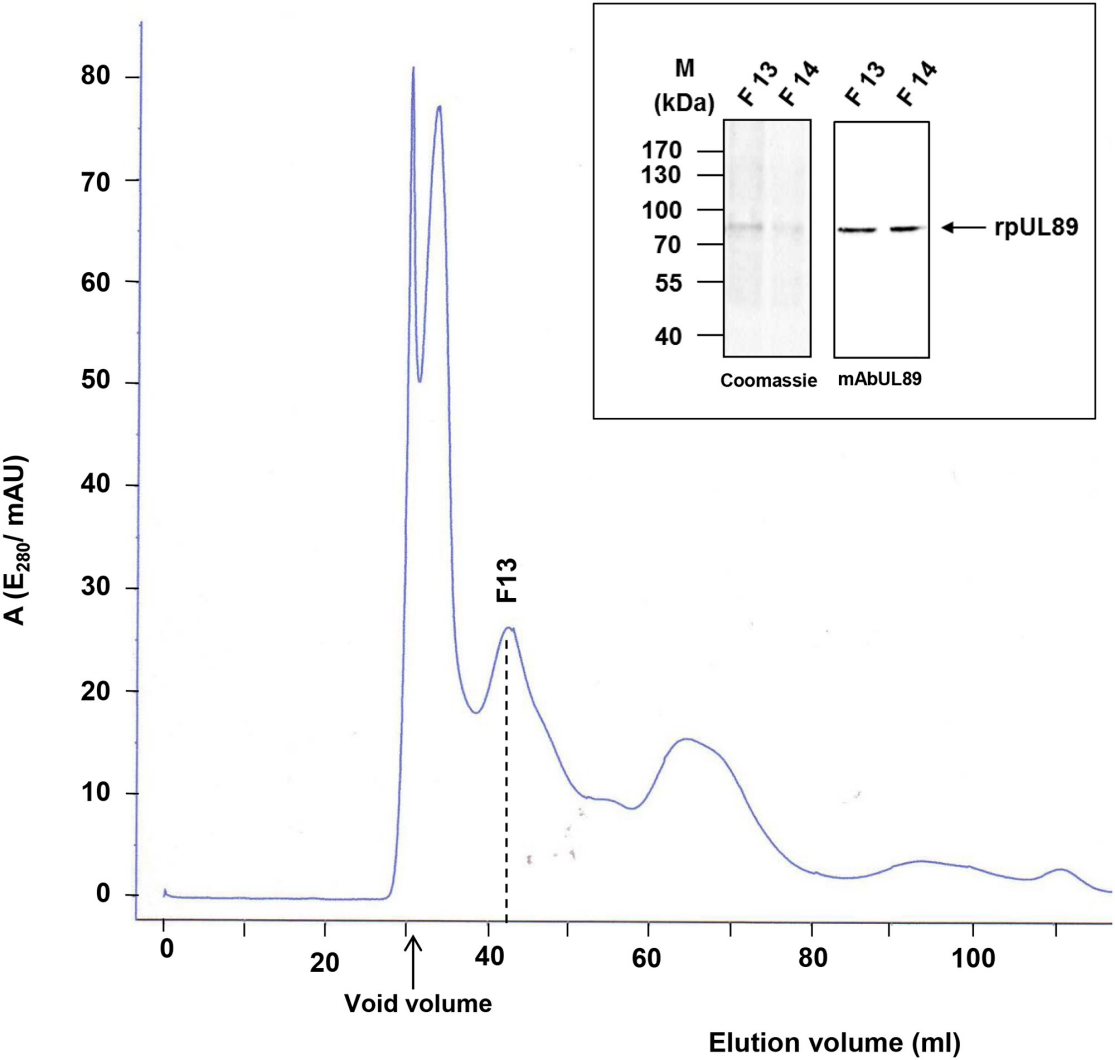
Purification of recombinant baculovirus expressed rpUL89. Gel permeation chromatography of rpUL89. The elution profile has 4 peaks. Coomassie-staining and immunoblot with monospecific mAbUL89 of fractions 13 and 14 are shown in the box. Molecular mass markers are indicated on the left, the position of pUL89 on the right.

To obtain further insights into the structure recombinant baculovirus-pUL89-infected High Five cell extracts were purified with cation-exchange chromatography followed by gel permeation chromatography (Fig 6). Aliquots of the rpUL89 containing fraction after ion-exchange chromatography were subjected to gel permeation chromatography.

The second peak after the void volume in the elution profile was assayed for protein content using Coomassie staining and immunoblotting with monospecific antibody mAbUL89. Fraction 13 corresponds to the molecular mass of a pUL89 monomer (Fig 6, integrated box), and was used for single particle analysis. Electron microscopy was carried out with negatively stained specimen. More than 1.500 particles of rpUL89 were selected and sorted into class averages (S5 Fig).

The asymmetric triangle is presented as an assurance that a sufficient number of different projections was available for the analysis with each dot presenting a different projection. A full triangle means that all principal and intermediate projections were available for analysis. The asymmetric triangle confirms that rpUL89 can assume many different orientations on the support film (S6A Fig) and that the corresponding projections are appropriately represented in the 3D reconstruction. Fourier shell correlation shows a converging iterative refinement and self-consistency of data to ~3nm at 0.5 σ (S6B Fig). With 0.5 nm/pixel at the specimen level, the data are only interpreted to a resolution far away from Nyquist and there is no danger of undersampling. The slight upward trend observed towards higher spatial frequencies is a result of the tight mask used for boxing the single molecules. This was, however, necessary in order to achieve an appropriate separation between the particles given their high packing density.

**Fig 7:**
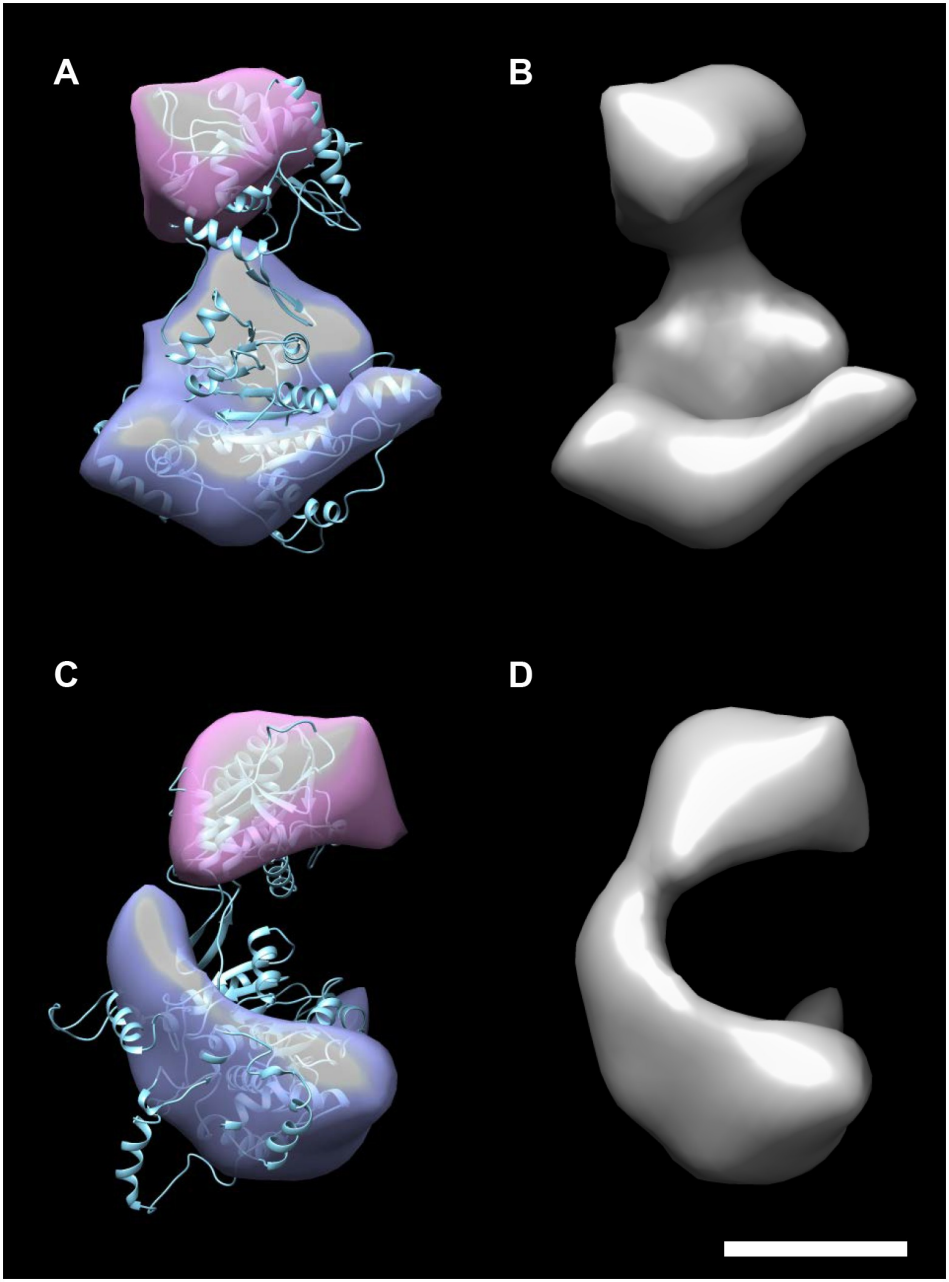
3-D reconstruction from single particle analysis of rpUL89. Surface-rendered presentations of the rpUL89 3D reconstruction are shown face-on from the front (A,B), and side-on to the right (C,D) with (A, C) and without (B, D) the predicted structure of pUL89. The scale bar corresponds to 5 nm. The magenta colored segment represents the C-terminal domain and the blue colored the N-terminal domain of pUL89.

The surface rendering of the 3-D reconstruction reveals a curvilinear monomer delineating two major protein domains linked by a minor density (Fig 7). By using the program Chimera segments were automatically created. The segment map correspond to the N- and C-terminal domains (Fig 7). A comparison of the 3-D surface structure obtained by single particle analysis with the Phyre2 [27] predicted pUL89 structure shows a reassuring degree of agreement. In order to obtain an objective, quantifiable measure for the merit of the positioning of the predicted structure of pUL89 within the 3-D envelope as determined by electron microscopy, a resolution-truncated 3-D map of pUL89 was generated from the Phyre2-predicted pdb file. The resolution was truncated at 3 nm to match the single particle reconstruction. After automated fitting and comparing the two 3-D maps in Chimera the correlation was 0.9014 speaking to the merit of the positioning.

## Discussion

Human cytomegalovirus replication leads to head-to-tail linked genomes, so called concatemers, which have to be cleaved by the terminase into unit-length genomes during packaging into capsids. HCMV terminase consists of the subunits pUL56 and pUL89 and a possible third component pUL51 [10, 11, 13, 20, 22]. HCMV is one of the most complex viruses and it seems to have evolved different functional arrangements of its terminase subunits compared to most herpesviruses and bacteriophages. In bacteriophages the large terminase subunit has a C-terminal nuclease and N-terminal ATPase activity, whereas the small subunit is only required for sequence specific binding. In contrast, both HCMV terminase subunits, pUL56 and pUL89, have nuclease activities, while pUL56 has an ATPase activity and also mediates sequence specific binding to packaging motifs.

The terminase subunit pUL89 is an essential component of the complex human cytomegalovirus DNA packaging process. This subunit seems to be mainly required for duplex nicking of the concatemers [22]. The HSV-1 homolog pUL15 is also critical for cleavage of the viral genome and packaging [6, 29]. HCMV pUL89 interacts with the terminase subunit pUL56, endowing the nuclease activity with sequence specificity, as well as with the portal protein pUL104 [30, 31, 32].

This study focuses on structure-function analyses of the terminase subunit pUL89. In view of its proposed function during packaging, experiments were performed to identify structural elements required for nicking and DNA binding of pUL89 based on a predicted structure originating from Phyre2 [27]. It was demonstrated that both terminase subunits are able to randomly nick DNA, if the nicking sites are at least 10 bp apart [22]. Sigamani et al. [26] reported a similar behavior using the C-terminal domain of HSV-1 homolog pUL15. Image analysis of single particles of pUL89 revealed a curvilinear structure in which two larger domains are connected by a less pronounced density. Sun et al. [24] demonstrated that bacteriophage T4 gp 17 can form a ring-like pentameric structure while the monomer had a structure similar to pUL89. Based on their function, terminases must have domains for DNA binding, translocation of DNA, interaction with other terminase subunits, and for nuclease activity.

Nadal et al. [25] reported that the nuclease motif of pUL89 resides at the C-terminus involving aa D463, E534 and D651. However, the data were based on a short C-terminal construct from aa418-674. In contrast to Nadal et al., we used the full-length protein (aa1-674) and found that E534 is not essential for cleavage, D651 has only a very low effect (only 10% of the DNA were ccc molecules; Fig 3B) and provide compelling evidence that a critical amino acid in full-length pUL89 is D463. However, one ought to be cautious when extending interpretations from an *in vitro* to an *in vivo* setting.

Furthermore, our data also show that a DNA binding motif of pUL89 is located at the C terminus (Fig 4). This motif corresponds to a postulated region with positively charged amino acids. Similar observations have been made with bacteriophage Sf6 terminase subunit gp2. Here protein mediated DNA binding is achieved via a linker by forming positive charged domains that contact the DNA phosphate backbone [33]. The different functions of pUL89 homologs in DNA packaging are illustrated in S2 Table [34–37].

From these data together with the structural information presented in this report, it emerges that those amino acids that participate in the function of this protein seem to be part of the C-terminus. We demonstrated that pUL56, the terminase subunit with ATPase activity, binds to specific DNA motifs, packaging motifs pac1 and pac2 [20]. Therefore, the specificity of DNA-binding and cleavage requires at least the interaction of both subunits pUL89 and pUL56. It is clear that more proteins are involved in DNA packaging due to the complexity of this process. We and others have shown that the proteins pUL104, pUL77, pUL93, pUL51, pUL52 are required for this process [11, 13, 32, 38–40]. PUL56 is characterized by a ring-like structure with a discontinuity on one side, and it has been previously suggested that the ring opening during DNA binding enabled by inherent conformational flexibility may be a prerequisite for its biological activity [22]. The same paper also showed what is deemed to be a pUL56-pUL89-DNA complex *in vitro* suggesting a highly synergistic process. While we identified the interaction domain of pUL89 with pUL56, the corresponding region in pUL56 has very recently been confirmed [31, 41]. Ligat et al. [37] demonstrated that the required amino acids (aa671-680) are located in the C-terminus of pUL56 and are essential for DNA replication. In order to endow the cleavage process with specificity, both proteins have to come into close proximity. How exactly the DNA is positioned relative to pUL56 when binding to pUL89 is currently not known.

Overall, in this report (i) a surface structure of the full-length HCMV terminase subunit pUL89 is presented, (ii) two key motifs in the terminase subunit pUL89, the nuclease and the DNA binding motifs are identified and (iii) invariant amino acids for both functions have been defined. While future studies will need to look into the formation and functionality of the terminase complex incorporating the DNA packaging protein and the portal protein, pUL77 and pUL104, the data presented here provided greater insight on the roles of individual amino acids in DNA packaging.

## Material & methods

### Cells and virus

Human embryonic kidney (HEK) 293T cells (ATTC, USA) were grown in Dulbecco`s minimal essential medium (DMEM) supplemented with 10% (v/v) fetal bovine serum (FBS), 2 mM glutamine and gentamicin (50 μg/ml). Insect cells 5B1-4 (High five; Life Technology) were grown in TC-100 medium supplemented with glutamine, gentamicin (60 μg/ml) and 10% (v/v) FBS. Recombinant baculovirus expressing pUL89 containing a N-terminal Anti-Xpress antibody epitope was generated by site-specific transposition of the expression cassette into the bacmid bMON14272 as described by Life Technology.

### Compound

2-Bromo-5,6-dichloro-(1-ß-D-ribofurnosyl)benzimidazole (BDCRB) were synthesized in the laboratory of Townsend [28]. Stock solutions 25 mM were obtained by dissolving the compound in dimethylsulfoxid. In the assays 0–15 μM BDCRB dissolved in PBS were used.

### Plasmid construction of pUL89 mutants

In order to determine the domains of pUL89 responsible for nuclease activity and DNA binding pUL89 mutants were generated.

According to the postulated protein structure mutants of pUL89 were generated by alanine substitution (pcDNA-UL89D463A, pcDNA-UL89E534A, pcDNA-UL89R544A, pcDNA-UL89H565A, pcDNA-UL89H571A, pcDNA-UL89D651A) using QuickChange site-directed mutagenesis with the following pairs of synthetic oligonucleotides as described in S1 Table (pcDNA-UL89 containing a His-tag at the N-terminus, served as template). All mutations were verified by DNA sequencing. The mutant containing plasmids were subjected to transfection as described below.

HEK 293T cells were transfected with the plasmids pcDNA-UL89 [30], pcDNA-UL89D463A, pcDNA-UL89E534A, pcDNA-UL89R544A, pcDNA-UL89H565A, pcDNA-UL89H571A or pcDNA-UL89D651A using TurboFect (Fermentas) according to the manufacturer’s protocol. At 48 h after transfection, cells were harvested and subjected to protein purification.

Protein concentration of cell extracts were determined by Pierce BCA Protein Assay Kit (Thermo Fisher Scientific) and extracts were diluted to a concentration of 1 mg/ml with buffer A (50 mM Tris/HCl (pH 7,4), 100 mM NaCl, 5 mM MgCl_2_, 10 mM imidazole, 0,05% Tween 20, 0,1 mM PMSF). Cell extracts from cells transfected with UL89 wild-type or UL89 mutant were purified using His Mag Sepharose Ni (GE Healtcare). After incubation of one hour, magnetic beads were washed with buffer A including 60 mM, 150 mM and 250 mM imidazole. Elution was performed with buffer A and 500 mM imidazole. The fractions were pooled and a second purification step to remove imidazole were carried out using a PD midi-Trap G-25 column (GE Healtcare) and buffer B (10 mM Tris/HCl (pH 7.4), 50 mM NaCl, 10 mM MgCl_2_, 1 mM DTT, 0,1 mM PMSF). Fraction were pooled and concentrated using an Amicon Ultra Centrifugal Filter (50K) (GE Healtcare). Protein concentration were calculated by Bradford assay.

### Nuclease Assay of purified pUL89

*In vitro* Nuclease Assays were modified [20] using 0,5 μM of purified pUL89 or mutant proteins and 600 ng of pUC-aseq. The plasmid pUC-aseq contains one *a* sequence including packaging motifs (pac1 and pac2). The region 730bp-950bp from the plasmid pON205, kindly provided by E. Mocarski, was inserted into the pUC18 vector [38]. Addition of the terminase inhibitor BDCRB (0–15 μM) was used to characterize the nuclease activity. The samples were incubated at 37°C for 60 min in a total volume of 10 μl nuclease buffer (10 mM Tris-HCl, pH 7.5, 10 mM MgCl_2_, 1 mM DTT, 50 mM NaCl 0,1 mM PMSF). After addition of proteinase K (final concentration: 1 μg/μl) the samples were incubated for 60 min at 65 °C and subjected to 0,8% (w/v) agarose gel electrophoresis. Different DNA forms can be distinguished. Nicking of one strand of the covalently closed circular (ccc) DNA leads to one open circular DNA molecules. Further nicking of the other circular DNA strand leads to linear DNA molecules and even smaller fragments.

### Colorimetric detection of the DNA-binding activity of purified pUL89

The colorimetric detection assay is based on two strong and specific interactions: (i) binding of his-tagged pUL89 to nickel-coated microplate and (ii) binding of biotinylated DNA to avidin. The avidin is conjugated with horseradish peroxidase which catalyses a colorimetric reaction the detection of which enables quantification.

The assay was performed according to the description of Banasik and Sachadyn [42]. Briefly, the nickel-coated microplates (Thermo Fisher Scientific) were washed five times with washing buffer (WB; PBS, 1% Tween 20, 5 mM MgCl_2_) prior to immobilization of the his-tagged proteins (2.5 μg/well) in 100 μl WB. After 15 min incubation at room temperature, the wells were emptied, washed and loaded with unlabelled competitor DNA (20 nmol/well) or WB. After 10 min the overlay was removed, plates were washed and loaded with biotinylated 250 bp dsDNA (200 pmol/well) and incubated for 10 min. Unbound DNA was removed by washing and DNA binding was analysed by detection of immobilized protein with HRP-conjugated avidin. After 10 min incubation the wells were washed following loading of the chromogenic substrate TMB (3,3´,5,5´-tetramethylbenzidine). After additional 15 min the colour reaction was developed and read at 620 nm. The unlabelled competitor and the biotinylated 250 bp ds DNA contain the DNA fragment 877-1126 bp of the plasmid pUC18. Following oligonucleotides were used for PCR (5´-ATTTCACCCCCCCGCTAAAAACTCCGCCCCCCTGACGAG and 5´-Biotin-CGTGCACACAGCCCAGC or 5´-CGTGCACACAGCCCAGC).

### Antibody against pUL89

HCMV pUL89 specific human monoclonal antibody (mAbUL89), kindly provided by S. Jonjic [11]. The specificity of mAbUL89 was determined using an immunoblot with cell extracts from mock- or infected HELF, HEK 293T, HEKT 293T transfected with pcDNA-UL89 or purified pUL89. The immunoblot was reacted with mAbUL89 and subsequently with monoclonal antibody against GAPDH. The antibody mAbUL89 only reacted with infected HELF (Fig 2A, lane 2), pUL89 from transfected cells (Fig 2A, lane 4) or purified pUL89. (Fig 2A, lane 5). These observations demonstrate that mAbUL89 is a monospecific antibody against pUL89.

### PAGE and immunoblot analysis

Purified pUL89 or mutant proteins were purified as described above prior to solubilization in 4 x sample buffer (4% (v/v) ß-mercaptoethanol, 0.01% (w/v) bromophenol blue, 4% (w/v) glycerol, 4% (w/v) SDS, 0.2 M Tris-HCl (pH 6.8)), followed by separation on 10% (w/v) SDS-PAGE. Proteins were either stained with Silver/Coomassie or transferred to nitrocellulose sheets and subjected to immunoblot analysis as described previously [43]. The antibody mAbUL89 specific for pUL89 (1:1000; Fig 2A) was used as the primary antibody. For detection of primary antibody binding, horseradish peroxidase-conjugated anti-mouse F(ab´)_2_ fragments (1:2000 in PBS; Santa Cruz Biotechnology) and the ECL (Super Signal West Pico) reagent were used as recommended by the supplier (Pierce; Thermo Fisher Scientific, Germany).

### Silver staining

The silver staining was performed as described in Swain and Ross 1995 [44]. Briefly, after fixation in 40% Ethanol and 10% Acetic acid for 10 minutes the gel was washed in Aqua dest. To increase sensitivity the gel was incubated in 0,05% Glutaraldehyde, 0,01% Formalin and 40% Ethanol for 5 minutes. In the next step the gel was rinsed in 40% Ethanol and distilled water prior to incubation in 0,2 g/l Sodium thiosulfate. After two more washing steps the gel was incubated with the silver staining solution (0,1% Silver nitrate). Afterwards the developing step was carried out in 2,5% Sodium carbonate and 0,04% Formalin. The solution was changed prior to stopping the reactions with 5% Acetic acid. The gel was stored in distilled water.

### Modelling of pUL89 binding to DNA/ cutting DNA

Structure predictions were carried out with the Phyre2 web portal for protein modelling, prediction and analysis (in intensive mode) [27]. This program created in the first step a hidden Markov model (HMM) that is then scanned against a database of HHMs of proteins with known structure. The resulting first models are proceeded to further stepwise analysis [27]. Templates included the homologous proteins from bacteriophage T4 gp17 (PDB: 3EZK_B), thermophilic bacteriophage D6E large terminase (PBD: 5OE9_C), and HCMV pUL89 nuclease domain (PBD: 3N4Q_A) and bacteriophage Sf6 gp2 (PBD: 4IDH_A). For modelling the program UCSF Chimera [45] was used. The DNA model was docked onto pUL89 manually, taking particularly those amino acids into consideration that have been identified to be critical by mutagenesis and binding assays.

For visualization of the motif for DNA binding and motif with nuclease activity the PyMOL Molecular Graphics System, Version 2.0 Schrödinger, LLC was used.

### Protein purification of recombinant, baculovirus expressed pUL89

High five cells (4 x 10^8^) expressing the recombinant pUL89 [22] were harvested 48 h p.i. Sedimented cells were lysed in 50 ml cation exchange buffer (20 mM MES pH 6.5, 150 mM NaCl and cOmplete (Roche Life Science). Purification was essentially carried out as described by Thoma et al. (2006) [31]. Fractions containing the pUL89 were stored at –80 °C.

### Electron microscopy

Negatively stained specimens of purified pUL89 were prepared essentially according to Valentine et al. [46] using an aqueous solution of uranyl acetate (4% (w/v), pH 4.3) and omitting any fixation steps. The adsorption time was 20 seconds. Specimens were mounted onto 400 mesh copper grids and observed in a Jeol 1200 EX TEM operated at an accelerating voltage of 100 kV. Images were recorded at calibrated magnifications using an optically coupled 3 k slow scan CCD camera (model 15C, SIA, Duluth, GA) and Maxim DL imaging software, with the pixel size corresponding to 0.51nm for a nominal magnification of 50.000.

### Image analysis and 3D reconstruction

Image analysis was performed as described before [47]. Briefly, particle selection and particle averaging was performed using the EMAN software package. Images were bandpass-filtered and aligned by reference-free alignment [48]. Classes of particles representing identical projections were determined by multivariate statistical analysis and hierarchical ascendant classification. Cross-common lines Euler search performed on class averages generated initial models, and angular refinement iteration was stopped when no further improvement in the statistics was observed. Self-consistency of the data was assessed using Fourier shell correlation (FSC) [49,50]. All 3D structures were visualized using the UCSF Chimera software package [51].

### Sequence alignment

In order to identify conserved region of the loop region of HCMV pUL89 a multiple sequence alignment with HSV-1 UL15, Bacteriophage T 4 gp17, RCMV E89 and CCMV TerL using the program Clustal Omega [52] (EMBL-EBI; https://www.ebi.ac.uk/Tools/services/web_clustalo/toolform) were performed.

### 1D gel quantification

In order to quantify the results from the nuclease activity assays we use the software Phoretix 1D (BioSystematica). The Phoretix 1 D is an advanced 1-D gel analysis software that can compare patterns within a single gel. This 1 D gel quantification software included background subtraction, normalisation, Gaussian fit bands and quantity calibration.

### Statistical analysis of DNA binding

All experiments were performed a minimum of three times. Data were expressed as mean ± standard deviation (SD). The results obtained from Students t-test were used to calculate significance of the DNA binding assays. A *p* value of ≤ 0.05 was considered significant.

## Supporting information

S1 Fig Proteins used for Phyr2 *in silico* analysis. The key principle of protein structure prediction of Phyre 2 are (i) that protein structure is more conserved in evolution than protein sequence and (ii) that there is evidence of a relatively small number of unique protein folds in nature (1.000–10.000). The protein structure prediction of pUL89 was based on the matching of its sequence to a library of known structures. The matched sequences are compared with the predicted 3-D structure of pUL89.
(TIF)

S2 Fig Comparison of the predicted structure of pUL89 with those of the homologous proteins. The ribbon representation of full-length pUL89 model (HCMV pUL89) was compared with structure of the nuclease domain (HCMV nuclease domain), deep sea thermophilic phage D6E TerL (phage D6E) and bacteriophage T4 gp17 (phage T4).
(TIF)

S3 Fig Multiple sequence alignment of HCMV pUL89, bacteriophage T4 gp17, HSV-1 UL15, RCMV E89 and CCMV TerL using the program Clustal Omega. Amino acids highlighted in gray are located in the loop region and involved in the putative DNA binding domain, while the aa highlighted in yellow are selected for mutagenesis.
(TIF)

S4 Fig Nuclease activity assays with different concentrations of pUL89. (A) Lane 1, 600 ng pUC-aseq; lane 2, incubation with restriction endonuclease Hind III, lane 3 incubated with 0.1 μM pUL89, lane 4, incubated with 0.2 μM pUL89; lane 5, incubated with 0.3 μM pUL89; lane 6, incubated with 0.4 μM pUL89; lane 7, incubated with 0.5 μM pUL89; lane 8, incubated with 0.6 μM pUL89; lane 9, incubated with 0.7 μM pUL89; lane 10, incubated with 0.8 μM pUL89; lane 11, incubated with 0.9 μM pUL89; lane 12, incubated with 1.0 μM pUL89; lane 13, incubated with 1.5 μM pUL89; lane 14, incubated with 2.0 μM pUL89. (B) Lane 1, 250 ng linearized pUC-aseq; lane 2, incubation with 0.5 μM pUL89, lane 3, incubated with 1.0 μM pUL89, lane 4, incubated with 1.5 μM pUL89; lane 5, incubated with 2.0 μM pUL89; lane 6, incubated with 2.5 μM pUL89; lane 7, incubated with 3.0 μM pUL89; lane 8, incubated with 3.5 μM pUL89; lane 9, incubated with 4.0 μM pUL89; lane 10, incubated with 4.5 μM pUL89; lane 14, incubated with 5.0 μM pUL89. After incubation with DNA at 37°C, all probes were treated with proteinase K (final concentration 1 μg/μl). The arrows indicated three different plasmid DNA forms: circular covalently closed molecules (ccc), open circular molecules and linear forms. The quantifications were performed with the software Phoretix 1D (BioSytematica) and shown below the image.
(TIF)

S5 Fig Electron micrographs of negatively stained pUL89. Representative projections corresponding class averages and back projections in (A) and (B), respectively. The scale bar corresponds to 5 nm.
(TIF)

S6 Fig Angle distribution of particles within the asymmetric triangle and Fourier shell correlation (FSC). (A) The asymmetric triangle demonstrates that pUL89 assumes many different orientations on the support film and that the corresponding projections are appropriately represented in the reconstruction. (B) The FSC curves converge after 7 iterations and suggest self-consistent data to approximately 3 nm. The curves corresponding to iterations 8, 9 and 10 are drawn in blue. S is the abbreviation for spatial frequency.
(TIF)

S1 Table Oligonucleotide primers used for mutagenesis of pUL89. Mismatches are indicated in bold and underlined.
(TIF)

S2 Table Features of UL89 HCMV homologs. Amino acids required for ATPase activity are shown in orange, those for nuclease activity are shown in blue and for DNA binding are shown in red.
(TIF)
