# Supplementary figures and images for: Full-length human cytomegalovirus terminase pUL89 adopts a two-domain structure specific for DNA packaging
Source: PLoS Pathog. 2019 Dec 6;15(12):e1008175. doi: 10.1371/journal.ppat.1008175 (PMC6897398; doi:10.1371/journal.ppat.1008175)

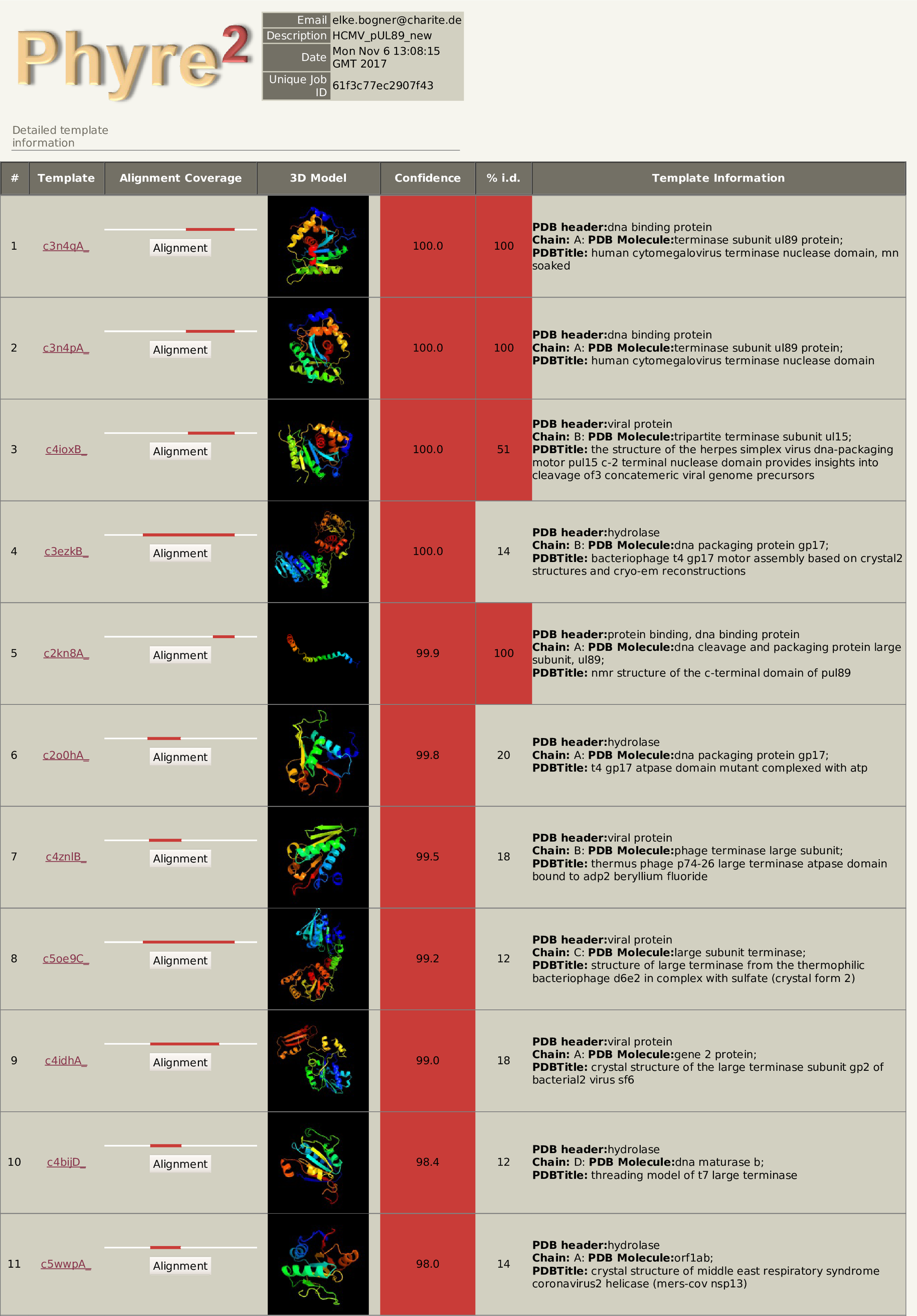

Supplement: S1 Fig — The key principle of protein structure prediction of Phyre 2 are (i) that protein structure is more conserved in evolution than protein sequence and (ii) that there is evidence of a relatively small number of unique protein folds in nature (1.000–10.000). The protein structure prediction of pUL89 was based on the matching of its sequence to a library of known structures. The matched sequences are compared with the predicted 3-D structure of pUL89. (TIF) [file ppat.1008175.s001.tif]

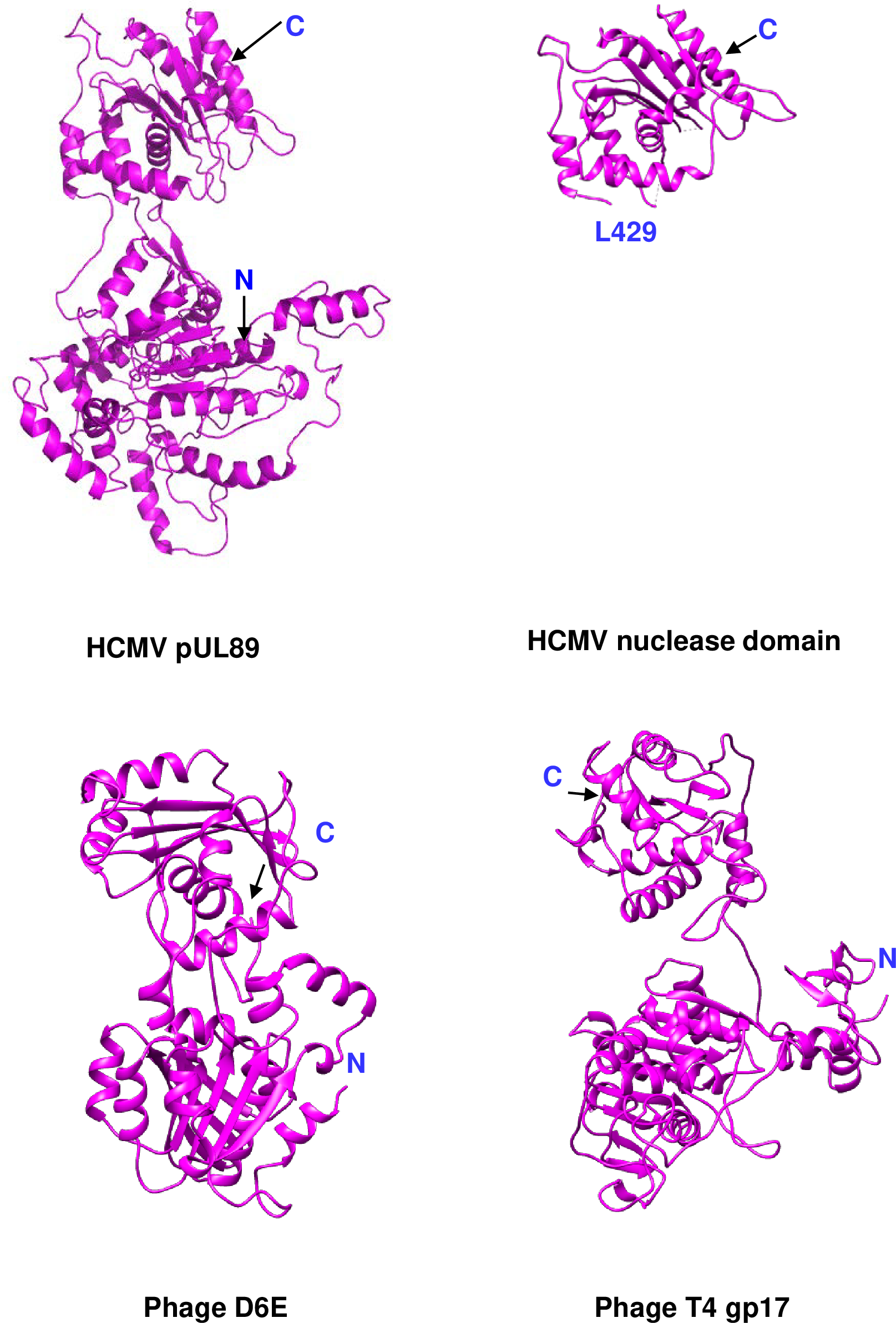

Supplement: S2 Fig — The ribbon representation of full-length pUL89 model (HCMV pUL89) was compared with structure of the nuclease domain (HCMV nuclease domain), deep sea thermophilic phage D6E TerL (phage D6E) and bacteriophage T4 gp17 (phage T4). (TIF) [file ppat.1008175.s002.tif]

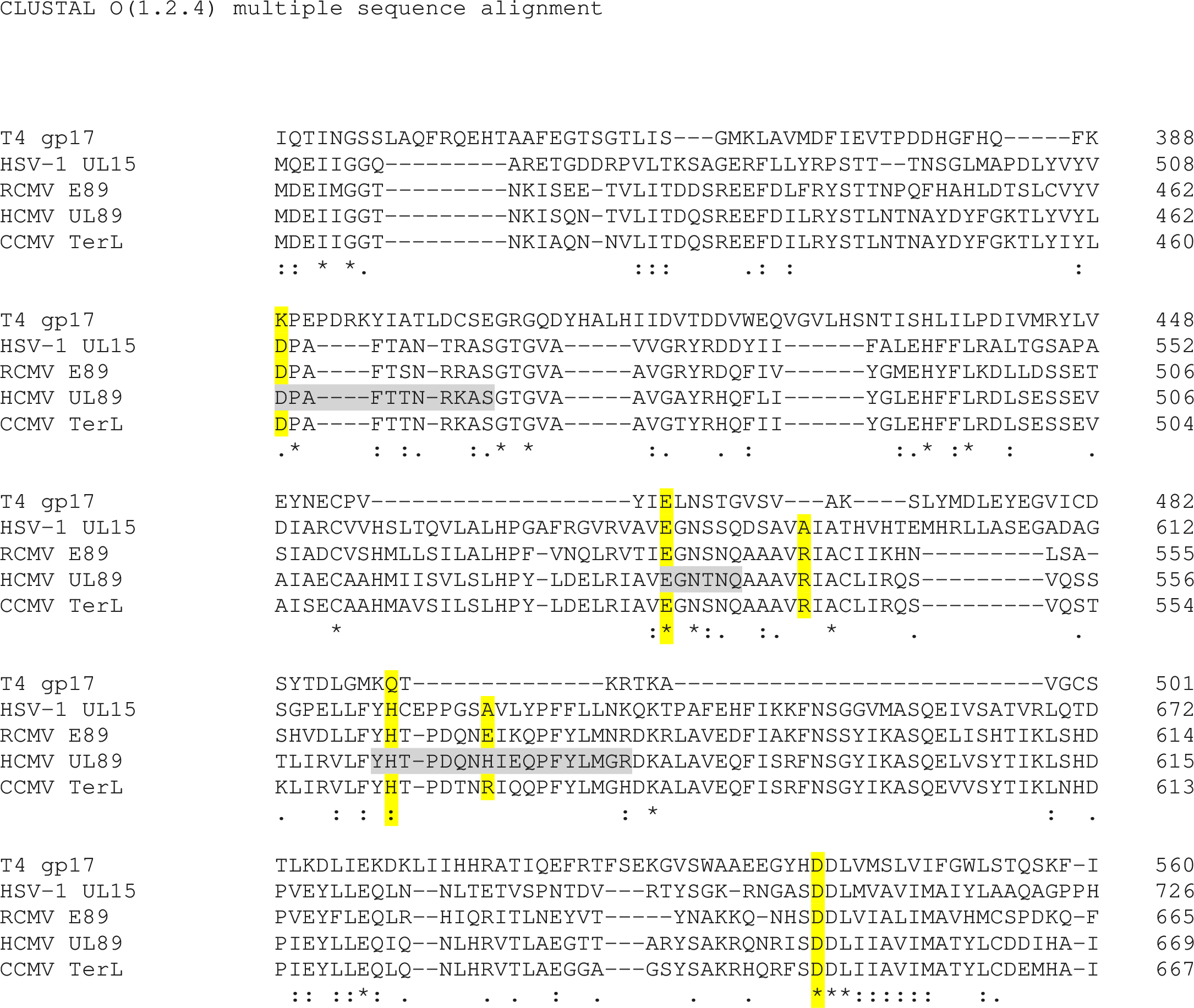

Supplement: S3 Fig — Amino acids highlighted in gray are located in the loop region and involved in the putative DNA binding domain, while the aa highlighted in yellow are selected for mutagenesis. (TIF) [file ppat.1008175.s003.tif]

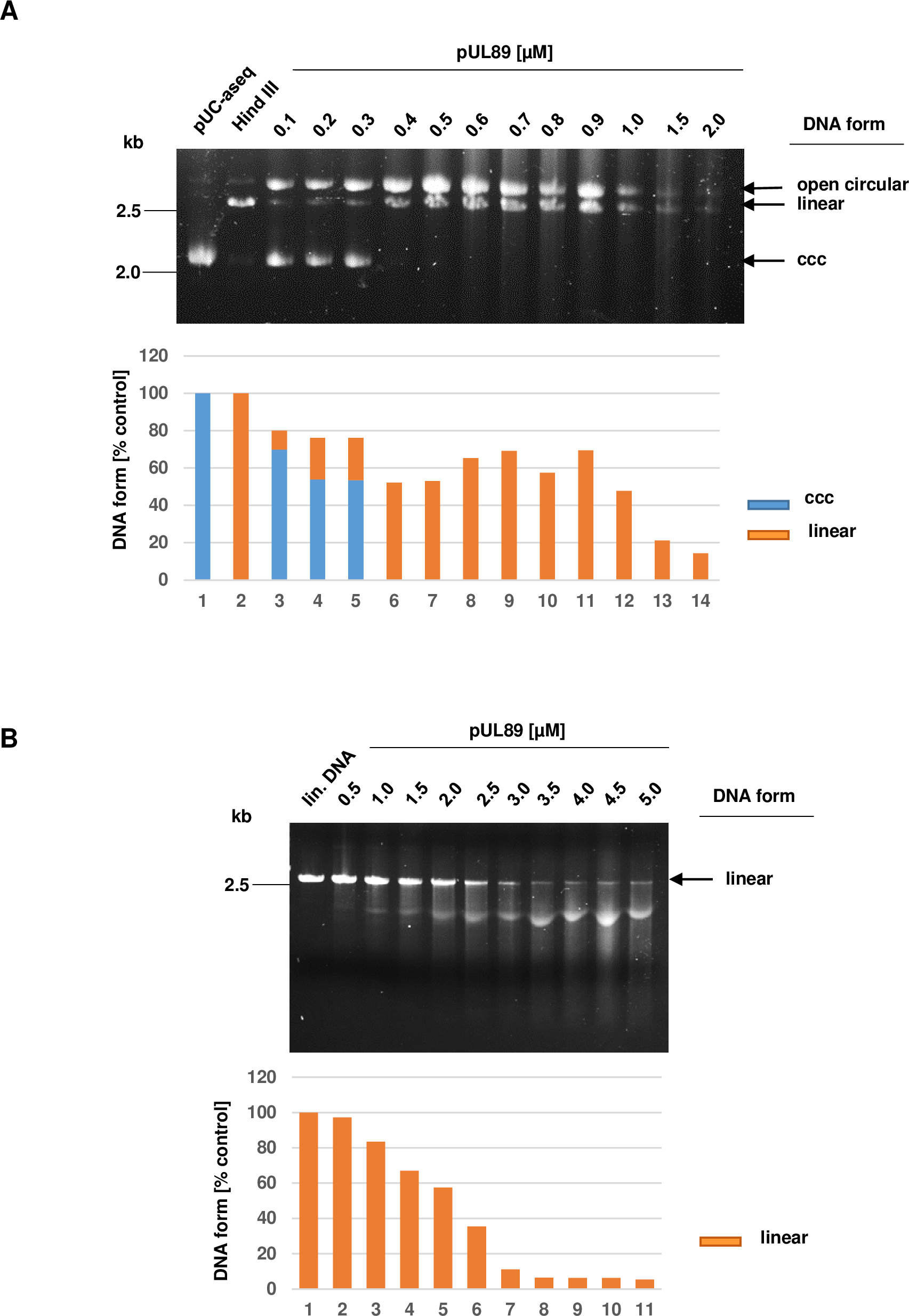

Supplement: S4 Fig — (A) Lane 1, 600 ng pUC-aseq; lane 2, incubation with restriction endonuclease Hind III, lane 3 incubated with 0.1 μM pUL89, lane 4, incubated with 0.2 μM pUL89; lane 5, incubated with 0.3 μM pUL89; lane 6, incubated with 0.4 μM pUL89; lane 7, incubated with 0.5 μM pUL89; lane 8, incubated with 0.6 μM pUL89; lane 9, incubated with 0.7 μM pUL89; lane 10, incubated with 0.8 μM pUL89; lane 11, incubated with 0.9 μM pUL89; lane 12, incubated with 1.0 μM pUL89; lane 13, incubated with 1.5 μM pUL89; lane 14, incubated with 2.0 μM pUL89. (B) Lane 1, 250 ng linearized pUC-aseq; lane 2, incubation with 0.5 μM pUL89, lane 3, incubated with 1.0 μM pUL89, lane 4, incubated with 1.5 μM pUL89; lane 5, incubated with 2.0 μM pUL89; lane 6, incubated with 2.5 μM pUL89; lane 7, incubated with 3.0 μM pUL89; lane 8, incubated with 3.5 μM pUL89; lane 9, incubated with 4.0 μM pUL89; lane 10, incubated with 4.5 μM pUL89; lane 14, incubated with 5.0 μM pUL89. After incubation with DNA at 37°C, all probes were treated with proteinase K (final concentration 1 μg/μl). The arrows indicated three different plasmid DNA forms: circular covalently closed molecules (ccc), open circular molecules and linear forms. The quantifications were performed with the software Phoretix 1D (BioSytematica) and shown below the image. (TIF) [file ppat.1008175.s004.tif]

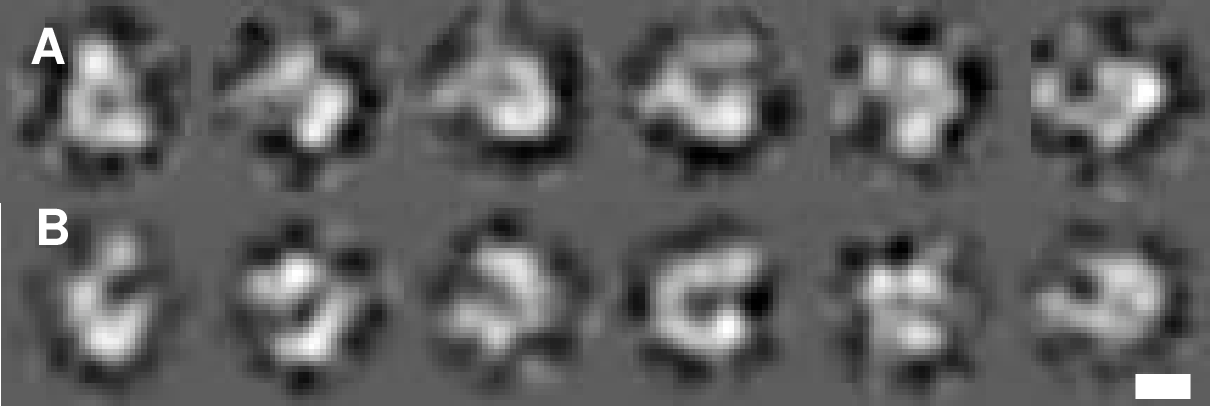

Supplement: S5 Fig — Representative projections corresponding class averages and back projections in (A) and (B), respectively. The scale bar corresponds to 5 nm. (TIF) [file ppat.1008175.s005.tif]

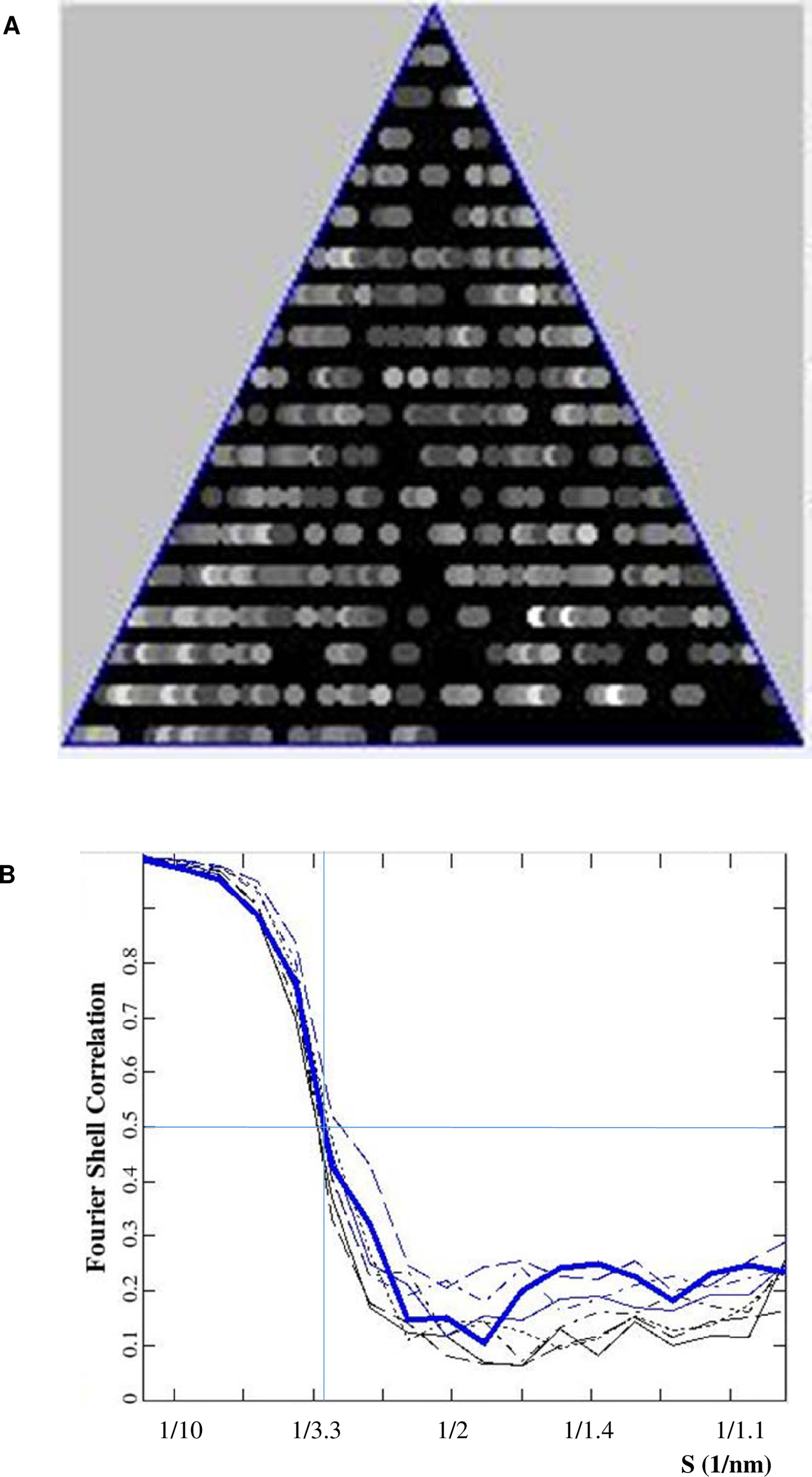

Supplement: S6 Fig — (A) The asymmetric triangle demonstrates that pUL89 assumes many different orientations on the support film and that the corresponding projections are appropriately represented in the reconstruction. (B) The FSC curves converge after 7 iterations and suggest self-consistent data to approximately 3 nm. The curves corresponding to iterations 8, 9 and 10 are drawn in blue. S is the abbreviation for spatial frequency. (TIF) [file ppat.1008175.s006.tif]

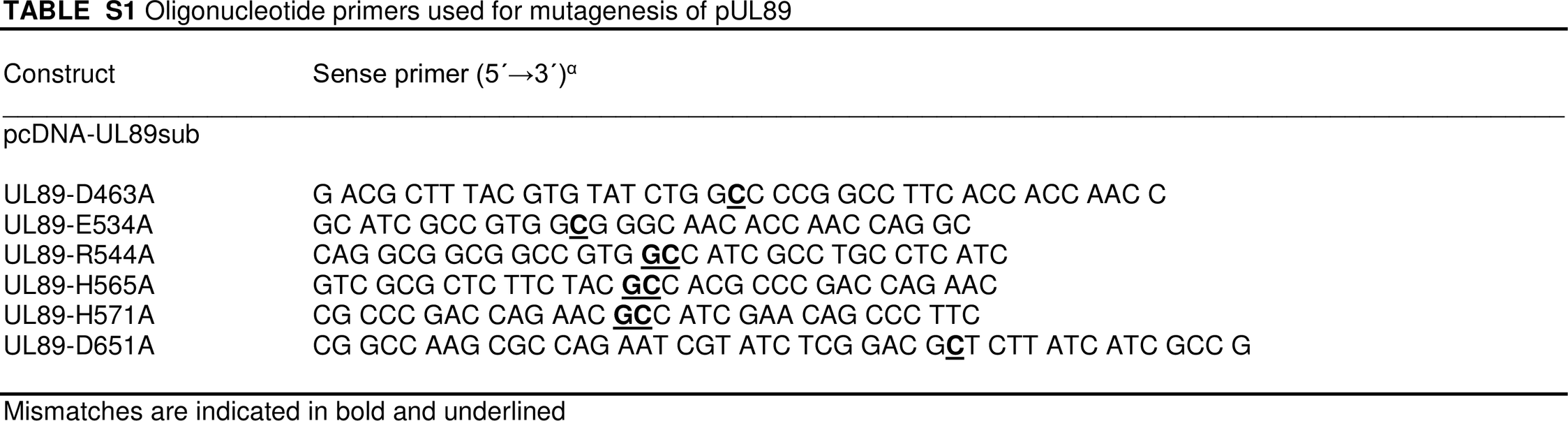

Supplement: S1 Table — Mismatches are indicated in bold and underlined. (TIF) [file ppat.1008175.s007.tif]

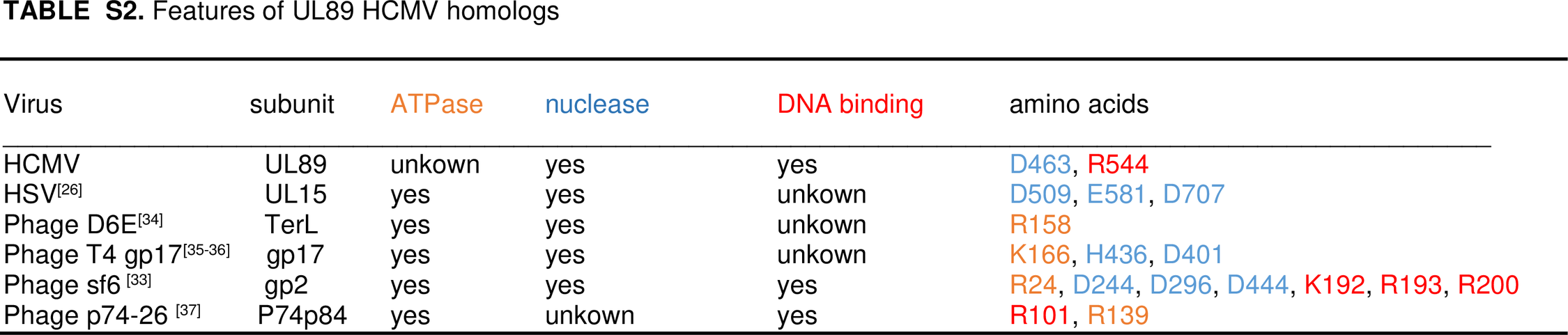

Supplement: S2 Table — Amino acids required for ATPase activity are shown in orange, those for nuclease activity are shown in blue and for DNA binding are shown in red. (TIF) [file ppat.1008175.s008.tif]
